# Albert Bruce Sabin: The Man Who Made the Oral Polio Vaccine

**DOI:** 10.3201/eid2803.204699

**Published:** 2022-03

**Authors:** Davide Orsini, Mariano Martini

**Affiliations:** University of Siena, Siena, Italy (D. Orsini); University of Genoa, Genoa, Italy (M. Martini)

**Keywords:** poliovirus, vaccine-preventable diseases, viruses, Sabin, poliomyelitis

## Who is this person?

Here is a clue: he made the oral polio vaccine.

Who is he?

A) Edward Jenner

B) Albert Bruce Sabin

C) Robert Koch

D) Jonas Edward Salk

Decide first. Then turn the page.

This is a photograph of Albert Bruce Sabin (1906–1993), the man who made the oral polio vaccine (Figure). Sabin’s name will always be associated with poliomyelitis, a disease that claimed millions of victims in the 20th century, particularly among children. At the beginning of the polio eradication initiative in 1988, the World Health Organization (WHO) estimated that ≈350,000 cases of paralytic polio were still occurring each year and that in the prevaccine era ≈650,000 cases occurred each year.

**Figure:**
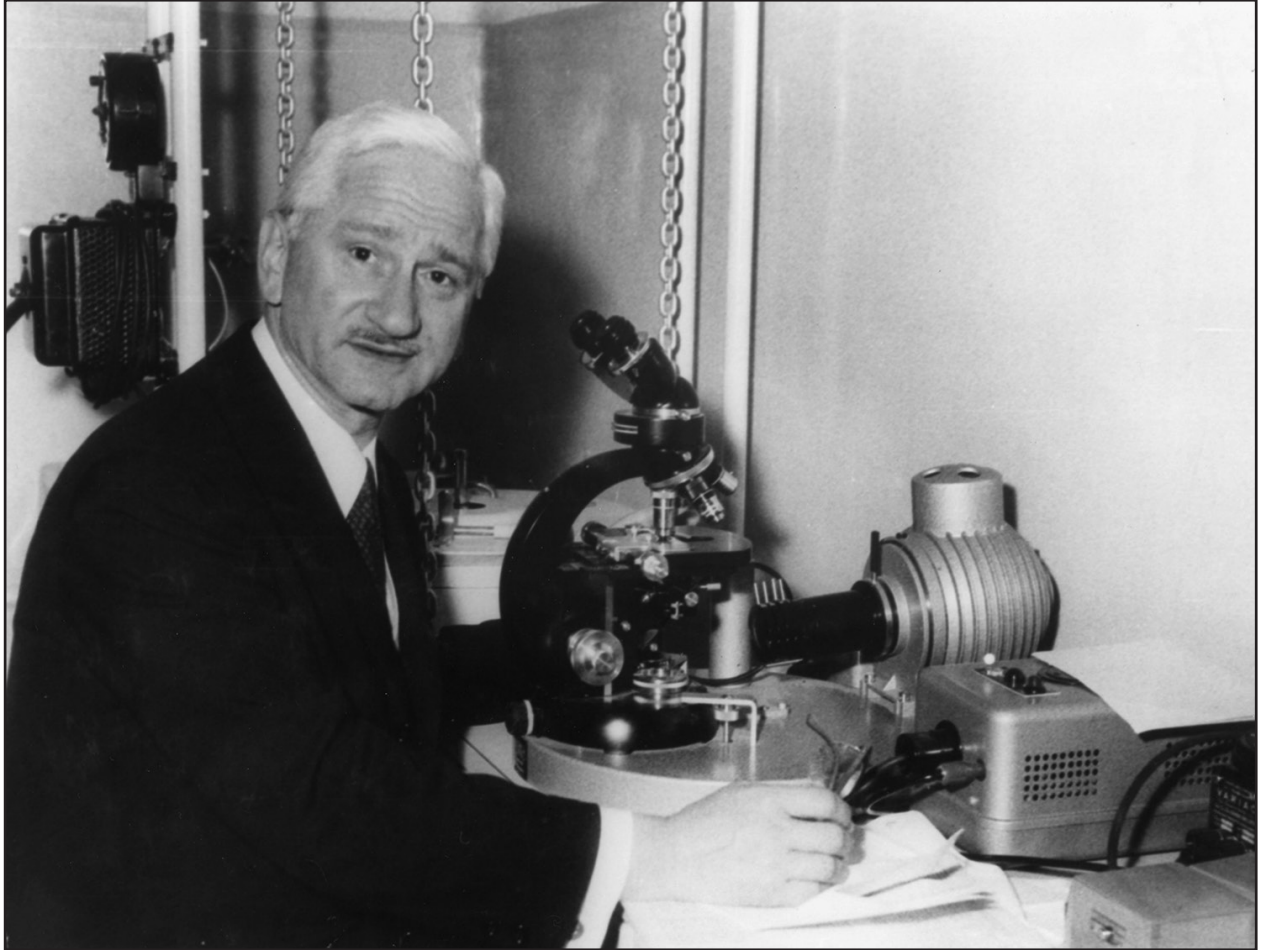
Albert Bruce Sabin

It was his mentor, William Hallock Park, famous for his research into a diphtheria vaccine, who in 1931 first urged Sabin to study poliomyelitis. In that year, Sabin had just finished his medical studies and polio was again raging in the United States, causing ≈17,000 cases of disease annually.

Indeed, in the first half of the 20th century, recurrent epidemics of poliovirus broke out during the hot season, striking thousands of children >2 years of age and also several adults. The most severely affected persons died or were left paralyzed, deformed, and unable to breathe outside an iron lung. Although a relatively low percentage of those affected died, millions of survivors carried the marks of the disease for the rest of their lives.

As the number of cases continued to grow, reaching a peak of ≈3,100 deaths and ≈21,000 cases of paralysis in 1952 in the United States, terror spread through a whole generation. According to the virologists, the only hope was to produce a vaccine. However, the first 2 attempts, in 1934 and 1935, failed dismally, resulting in a large number of victims.

In the middle of the 1930s, Sabin continued his studies on poliovirus, the etiologic agent of poliomyelitis, and in 1939 he realized that it was not a respiratory virus but an enteric virus that lived and multiplied in the intestine. Moreover, he was able to demonstrate that contagion occurred through both the respiratory route from coughing and sneezing and the enteric route from fecal contamination.

Starting from this important discovery, Sabin set out to create a vaccine against poliovirus. The pathway was not simple, and he ran into numerous obstacles, not all of which were of a scientific nature. Moreover, his work led him to clash with Jonas Salk in one of the most celebrated scientific challenges of the 20th century.

Salk, a researcher at the University of Pittsburgh, created his inactivated polio vaccine (IPV) during 1952–1953. The vaccine contained wild polioviruses of all 3 serotypes that had been killed by means of formaldehyde; when injected intramuscularly, the vaccine elicited the production of antibodies, rendering recipients immune to the disease. On April 12, 1955, it was proclaimed that the battle against poliomyelitis had potentially been won thanks to Salk’s vaccine. Unfortunately, however, 2 defective lots of the vaccine produced by Cutter Laboratories, a pharmaceutical company in Berkeley, California, contained residual live polioviruses, causing the so-called Cutter incident. In total, 192 paralytic polio cases occurred among vaccinated children and their family and community contacts, of whom 11 died. The government temporarily suspended the vaccination program until it was determined that Cutter vaccine should be permanently withdrawn and IPV from other manufacturers could be reinitiated safely.

Meanwhile, at the University of Cincinnati, Sabin was also at work on his vaccine. His approach, however, was completely different; he aimed to create a live attenuated vaccine for oral administration. This process marked the advent of the oral polio vaccine (OPV).

Sabin created his vaccine at the Children’s Hospital in Cincinnati, where he subsequently tested it on 10,000 monkeys and 160 chimpanzees, as well as on himself, on his daughters, and on young volunteers recruited from among the inmates of the federal prison of Chillichote in Ohio. However, because the painful memory of the Cutter incident was still fresh and especially because the commercial stakes were high, the US government did not consent to large-scale field testing.

Subsequent testing of Sabin’s vaccine was therefore carried out in the Soviet Union. During 1959–1961, millions of children received Sabin’s vaccine (77 million in the Soviet Union alone). These early vaccination campaigns yielded very good results, just as the 1958 campaign had done in the Belgian Congo, a region that had been severely afflicted by the virus.

The mass vaccination campaign in the Soviet Union demonstrated high vaccine effectiveness and resulted in licensure of OPV in the United States in 1961. Subsequently, in the United States, OPV rapidly replaced IPV during the 1960s as the vaccine of choice. OPV was preferred over IPV because it induced both systemic and intestinal immunity, was easier to administer, and was less expensive than IPV. The main drawback of OPV is that, very rarely (in 1 case out of ≈750,000), Sabin viruses can mutate back to a more neurovirulent form and cause vaccine-associated paralytic polio.

In any case, Sabin’s vaccine, which was economical to produce and very easy to administer on a sugar lump to children, came to be used worldwide in the 1960s. The method of administration of the Sabin vaccine inspired the popular song written by the Sherman brothers, featured in the film *Mary Poppins* whose refrain states, “Just a spoonful of sugar helps the medicine go down in a most delightful way.” In 1961, OPV was adopted in the United States, where, in the meantime, thanks to Salk’s vaccine, the spread of poliomyelitis had been markedly curbed.

Like Salk, Sabin did not patent his vaccine because he wanted it to be used as broadly as possible. “A lot of people insisted that I should patent the vaccine, but I didn’t want to do that,” he said. “It’s my gift to all the world’s children.” Thus, he refused to exploit the vaccine commercially, so that its price could be kept as low as possible.

Sabin’s preparation was subsequently adopted by WHO, becoming the mainstay of the worldwide vaccination campaign that enabled poliomyelitis to be eradicated from many countries. The last case in the United States was reported in 1979. In 1994, WHO certified the eradication of polio from the Americas, and in 2000 from 36 countries in the Western Pacific Region, including China and Australia. In 2002, Europe was certified polio-free, followed by the entire South-East Asia Region of WHO in 2014. After 2014, polio remained endemic in only 3 countries, Nigeria, Pakistan, and Afghanistan, until August 25, 2020, when Africa was declared totally polio-free.

Albert Bruce Sabin died in the hospital of Georgetown University in Washington on March 3, 1993, at the age of 86 years. Among the many accolades that he received, in 1970 he was awarded the National Medal of Science “for numerous fundamental contributions to the understanding of viruses and viral diseases, culminating in the development of the vaccine which has eliminated poliomyelitis as a major threat to human health.”
